# The Alaska Area Specimen Bank: a tribal–federal partnership to maintain and manage a resource for health research

**DOI:** 10.3402/ijch.v72i0.20607

**Published:** 2013-04-16

**Authors:** Alan J. Parkinson, Thomas Hennessy, Lisa Bulkow, H. Sally Smith, the Alaska Area Specimen Bank Working Group

**Affiliations:** 1 Arctic Investigations Program, Centres for Disease Control and Prevention, Anchorage, USA; 2 Bristol Bay Area Health Corporation, Dillingham, USA; 3 Joe Klejka, Yukon Kuskokwim Health Corporation; Mike Zacharof, Aleutian Pribilof Island Association; June Walunga and Emily Hughes, Norton Sound Health Corporation; Linda Clement, Metlakatla; Cristina Westlake, Maniilaq Health Corporation; Ileen Sylvester, Denise Dillard Southcentral Foundation; Terry Powell, Alaska Area Institutional Review Board; Joe McLaughlin, State of Alaska, Section of Epidemiology

**Keywords:** Biobanking, policy and procedures, Tribal and Federal Management

## Abstract

Banked biospecimens from a defined population are a valuable resource that can be used to assess early markers for illness or to determine the prevalence of a disease to aid the development of intervention strategies to reduce morbidity and mortality. The Alaska Area Specimen Bank (AASB) currently contains 266,353 residual biologic specimens (serum, plasma, whole blood, tissue, bacterial cultures) from 83,841 persons who participated in research studies, public health investigations and clinical testing conducted by the U.S. Public Health Service and Alaska Native tribal health organisations dating back to 1961. The majority (95.7%) are serum specimens, 77% were collected between 1981 and 1994 and 85% were collected from Alaska Native people. Oversight of the specimen bank is provided by a working group with representation from tribal, state and federal health organisations, the Alaska Area IRB and a specimen bank committee which ensures the specimens are used in accordance with policies and procedures developed by the working group.

In Alaska, access to banks of human biological specimens is important for research that improves human health (1–4). While there are numerous biobanks throughout the world that could be used to improve human health, access to these is often complicated by a number of ethical and legal conflicts related to the collection and use of specimens in health research (5–9). Although many indigenous peoples suffer from marked health inequities and may stand to benefit from health research conducted using banked specimens, past unethical and undesirable research practices have led to distrust and conflict between indigenous populations and researchers wishing to conduct research on banked specimens from those indigenous people. While these events have often complicated the development of ethical frameworks for biobank-based research using specimens from indigenous populations, they have led to discussions between researchers and indigenous peoples’ organisations on the establishment of guidelines that encourage indigenous community participation in health research (10–12). These guidelines have contributed to the development of various tribal codes that aim to control health research conducted within tribal jurisdictions (13, 14), and to the development of specific guidelines for policies regarding the collection and management of biological specimens collected during research involving indigenous peoples (15). The application of these guidelines to the operation and management of a biobank should prevent unethical and undesirable practices that have occurred in the past and help restore trust between indigenous peoples and researchers wishing to conduct research using banked specimens drawn from individuals from these communities.

This paper describes the formation of an Alaska Native tribal health and U.S. Federal partnership to develop policies and procedures that allow the secondary use of specimens collected from a largely Alaska Native population for the purposes of improving the health and well-being of that population.

## Methods

Extensive human health research has been conducted in Alaska for many years, particularly among Alaska Natives, to address health disparities and to improve the health of all Alaskans. Much of this research was conducted by agencies of the US Public Health Service: The Arctic Health Research Center (1948–1973), the Alaska Area Native Health Service (AANHS), The Indian Health Service (IHS) and the Arctic Investigations Program (AIP, 1973–present) of the Centers for Disease Control and Prevention (CDC). It has been the practice of these agencies to save and store biological specimens and associated personal identifiers from human subjects research (any biological material, including, but not limited to, serum, plasma, cell suspensions and other body fluids: cultures of etiologic agents, and tissues) that were not utilised during the original study. Testing of these previously collected biologic specimens (secondary use) has allowed the assessment of disease prevalence or contributed to the understanding of the clinical course of disease and thus has benefited the health and well-being of the Alaska Native population without the need to collect new specimens, thus saving time, resources and avoiding additional participant involvement and discomfort (1–4, 16–19)

**Fig. 1:**
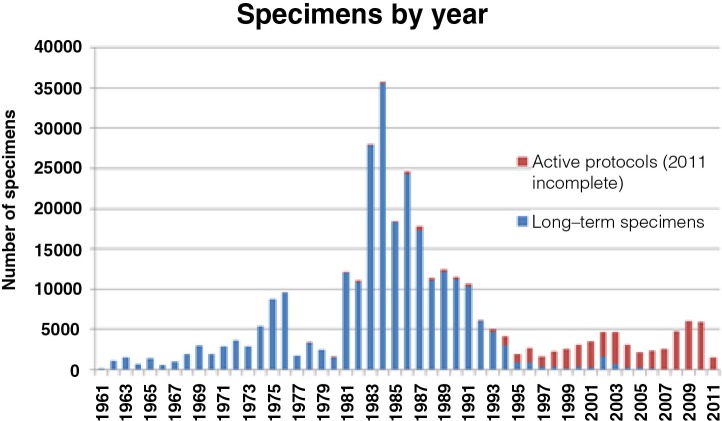
Alaska Area Specimen Bank: active and inactive specimens by year of collection. Figure shows numbers of specimens collected by year, including those specimens now inactive and available for secondary use (blue) and those collected under an active protocol and not available for secondary use (red).

As of 1 December 2011, the Specimen Bank contained 266,353 residual biologic specimens (serum, plasma, whole blood, tissue, bacterial cultures) from 83,841 persons who participated in research studies, public health investigations and clinical testing conducted in Alaska since 1961 (Fig. 1). Of these, 14,345 persons (17%) are now deceased. The majority (95.7%) are serum specimens, most specimens (77%) were collected between 1981 and 1994 and most (71%) were collected for viral hepatitis studies (18–23). The vast majority (85%) were collected from Alaska Native persons. Specimens are housed at the AIP in a specially designed walk-in –20°C freezer and stand-alone –70°C freezers. Approximately 98% are serum or plasma stored at −20°C. Bacterial cultures, tissues, cells and DNA account for 2% stored at −70°C. The majority of persons have only 1 specimen per collection date, although 5.6% of persons have specimens for more than 10 collection dates. Individual aliquots are computer catalogued by rack, layer and position linked to a central demographic file containing personal identifiers. All computerised data are stored in a password protected system with restricted access, housed at the AIP.

Prior to 1997 the Specimen Bank was jointly managed and operated by AIP and the IHS. Use of specimens stored and catalogued in the specimen bank was governed by the guidelines jointly developed by AIP and approved by the IHS and CDC Institutional Review Boards (IRB), and the Alaska Area Native Health Service research and publications committee (precursor to the current Alaska Area IRB). These guidelines subsequently contributed to the development of the IHS guidelines for collection and use of research specimens and policies and procedures for the Alaska Area Specimen Bank (15).

The Indian Self Determination Act (Public Law 93-638 (1975) has enabled Alaska Natives to assume responsibility for management of their health care system. Under this law many of the activities and services provided by the AANHS and IHS were transferred to Alaska Native Tribal Health organisations. This transfer of services together with increasing tribal interest and participation in conducting research benefiting Alaska Native people prompted a review in the composition, management and operation of the Specimen Bank.

## Results

A Working Group was established that is comprised of representatives from tribal health organisations, AIP, the State of Alaska/Division of Public Health, the Indian Health Service and the Alaska Area IRB. An early topic of discussion was the issue of ownership. The Working Group agreed that:

- The specimen bank would remain as a resource of the Alaska Native people held in trust to be used to benefit the health and well-being of Alaska Native people
- That individual specimens were the property of the study participant who provided consent to have that specimen banked for future study. The participant can request to have the specimen removed at any time.

The policies and procedures for the operation of the Specimen Bank were developed by the working group and were approved by both the Alaska Area and CDC IRBs and participating stakeholder tribal health organisations. (See Supplemental Material)

The policy and procedures specified the formation of a Specimen Bank Committee (The Committee). The Committee would manage the day-to-day operation of the Specimen Bank. The Committee would be comprised of AIP subject matter experts from administrative, computer/statistical and laboratory staff and would be chaired jointly by the Director of AIP (or designee) and by an Alaska Native Tribal Health organisation representative selected from the working group. The Committee meets monthly and ensures that the specimens are used in accordance with the policies and procedures defined by the working group. The Committee manages protocols; ensures that appropriate approvals, consents and renewals are obtained; communicates with principal investigators regarding specimen collection, shipping and banking, to ensure high quality of specimens and information; ensures privacy and confidentiality; and oversees accessioning, data management, quality control and quality assurance. The Committee oversees specimen withdrawal during a study and removal of specimens at the end of study, or at the individual study participant's request. The Committee makes periodic reports on Specimen Bank activity to the working group, including changes to the policies and procedures governing the specimen bank and the IRB approval process; updates on research protocols specifying specimen collection and storage that are pending IRB approval, renewal or closure; and updates on progress of ongoing projects that require specimen collection and storage.

While several guidelines have been published that describe how to conduct health research in partnership with indigenous peoples organisations (10–12), and how to manage specimen collection, storage and secondary use (13–15, 24), we believe this is the first description of an operational system that has engaged an indigenous community in the process of developing policies and procedures for the management of a Specimen Bank. The policies and procedures were developed in partnership with regional Alaska Native tribal health representatives and resulted in the formation of an advisory working group that would provide oversight of the specimen bank, and establish a forum for ongoing discussion and resolution of concerns and issues related to use of banked specimens.

**Fig. 2:**
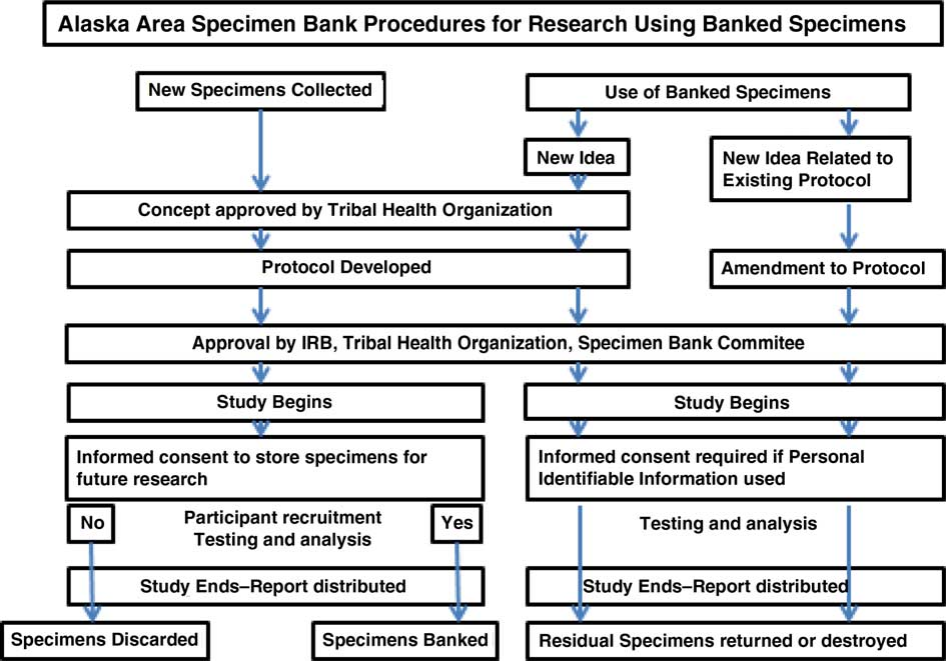
Alaska Area Specimen Bank procedures for research using banked specimens.

In brief, the policy and procedures specify that any research that requires the collection of specimens for storage or use of banked specimens must be approved by the tribal health organisation(s) that represents the potential study participants (Figure 2). This is facilitated by the submission of a letter of concept submitted by the researcher to the appropriate tribal health organisation and if approved would be followed by submission of a detailed protocol describing the proposed research both to the tribal health organisation and the Alaska Area IRB. In order for specimens to be added to the Specimen Bank, they must have been obtained using a research protocol approved by the Alaska Area IRB. A key element of the research protocol is a description of the informed consent process which must provide the participant with the options of participating in the proposed research, and must document agreement to have any remaining specimen and identifying information (name, date of birth, village of residence) stored in the bank for future research, or to participate in the proposed research without having specimens and identifying information stored long term for future research. Depending on the study protocol, approval for secondary use of the specimens may contain restrictions such as limiting the research to infectious diseases or to prohibit human genetic studies.

Researchers may request to use banked specimens for purposes either related or unrelated to the original study goals and objectives. If use of specimens is required for research that is related to the original study goals and objectives and the study protocol is still active under an IRB, an amendment to the original study protocol is required and must be approved by both the tribal health organisation(s) that approved the original study and the Alaska Area IRB. If use of specimens is not related to the original study goals and objectives, then a new research protocol approved by the Alaska Area IRB and tribal health organisation(s) is required. Any new study that requests access to banked specimens linked to a participant's identifying information will require an additional consent from the study participant. Secondary use of stored specimens not linked to a participant's identifying information (i.e. anonymous testing) does not require the additional consent from individual study participants, but does need the approval of the appropriate tribal health organisation.

Once a protocol is approved, The Committee has the responsibility of notifying the researcher of the conditions under-which specimen collection, testing and banking will occur; the limitations with regard to sharing of specimens with other investigators; and the timeline to return or destroy unused specimens at the completion of the study. When the study ends, the researcher returns documents confirming destruction of any unused specimens. Remaining banked specimens from the study will be either discarded or return to long-term storage in the Specimen Bank as specified by each individual study participant's consent. The Committee ensures accountability by providing oversight of the collection and use of specimens according to the wishes of the research participant as expressed in the informed consent document, and adherence to the policies and procedures that will guide the operation of the specimen bank.

## Discussion

The policy and procedures developed for the Alaska Area Specimen Bank was modelled on current federal guidelines for human subject research and the use of specimens that are derived from human subjects in clinical and research studies (24). Such guidance specifies the need for IRB oversight of any federally funded tissue repository and requires written study participant consent for the collection and use of tissue samples in a specific research study. While current federal regulations do not specifically address the use of those specimens and data after the study is completed, it is clear that research involving identifiable specimens is subject to regulatory oversight. However, it is not clear as to whether or not a tissue repository should be considered research. Researchers often use old specimens in new research without additional consent or IRB approval. In contrast, secondary use of specimens stored in the Alaska Area Specimen bank requires re-consent if specimens are linked to a study participant's identifying information, but would not require additional consent if specimens used are not linked to identifying information. However, proposed changes to the federal regulations would strengthen informed consent protections involving “secondary use” of specimens and data (25) collected either for clinical or research purposes. The new rule would now require a person to provide written consent for future “open ended” research use of specimens.

The tribal–federal partnership established for the co-management of the Specimen Bank fits well with the Alaska Native Tribal Health system and is also consistent with tribal sovereignty and self-determination in areas of human health research. This partnership created a management structure that includes both the cultural expertise of tribal leaders with the scientific and public health expertise of state, tribal and federal partners. This benefits not only the management of Alaska Area Specimen Bank, but also provides a forum for further discussion of proposed research involving Alaska Native communities. This ensures ongoing review and refinement of policies and procedures governing the operation of the specimen bank. Areas of ongoing discussion include: the use of historical specimens without contemporary informed consent; the use of specimens from deceased persons; and the potential expansion of the policy and procedures to include banking of pathological tissue samples, paraffinised tissue blocks, cells, and human DNA.
